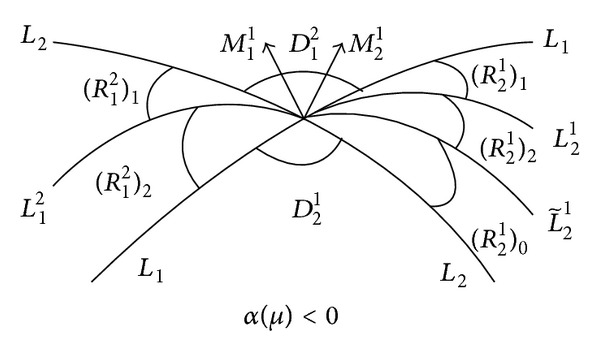# Bifurcations of Nontwisted Heteroclinic Loop with Resonant Eigenvalues

**DOI:** 10.1155/2014/716082

**Published:** 2014-04-16

**Authors:** Yinlai Jin, Xiaowei Zhu, Zheng Guo, Han Xu, Liqun Zhang, Benyan Ding

**Affiliations:** ^1^School of Science, Linyi University, Linyi, Shandong 276005, China; ^2^School of Mathematics Science, Shandong Normal University, Jinan 250014, China

## Abstract

By using the foundational solutions of the linear variational equation of the unperturbed system along the heteroclinic orbits to establish the local coordinate systems in the small tubular neighborhoods of the heteroclinic orbits, we study the bifurcation problems of nontwisted heteroclinic loop with resonant eigenvalues. The existence, numbers, and existence regions of 1-heteroclinic loop, 1-homoclinic loop, 1-periodic orbit, 2-fold 1-periodic orbit, and two 1-periodic orbits are obtained. Meanwhile, we give the corresponding bifurcation surfaces.

## 1. Introduction and Hypotheses

With the development of nonlinear science and the deep study of chaotic phenomenon, many experts have been interested in the research on the bifurcation problems of homoclinic and heteroclinic loops for high-dimensional nonlinear dynamical systems (see [1–5] and references therein). Compared with the bifurcations of homoclinic and heteroclinic loops for planer systems, the obtained results have been not rich because of the complexity and the lack of methods. In 1990, Chow et al. studied the codimension 2 homoclinic bifurcation under nondegenerate condition at resonant eigenvalues in [6]. The traditional methods to construct Poincaré maps were adopted to later research. In 1998, Zhu studied the bifurcation problems of nondegenerated homoclinic loops for high-dimensional system $\dot{z}=f\left(z,\alpha \right)+\epsilon g\left(z,\mu ,\epsilon \right)$ in [7] and the bifurcations of nondegenerated heteroclinic loops for high-dimensional system $\dot{z}=f\left(z\right)+g\left(z,\mu \right)$ in [8]. The methods used in [7, 8] were by generalizing the Floquet method to build the local coordinate systems and Poincaré map. The papers [7, 8] used the inherent characteristic values to describe the bifurcation surfaces and phenomena so that the results had the practicability and maneuverability.

In [9], the authors studied the bifurcations of rough heteroclinic loops with two saddle point. In [10], Tian and Zhu studied the bifurcations of fine heteroclinic loops with two saddle points. The heteroclinic loops studied in [9, 10] were both nontwisted and with no resonant eigenvalues.

In this paper, we study the bifurcation problems of heteroclinic loop with resonant eigenvalues for high-dimensional system $\dot{z}=f\left(z\right)+g\left(z,\mu \right)$. By simplifying the method in [7, 8], we use the foundational solutions of the linear variational equation of the unperturbed system along the heteroclinic orbits to establish the suitable local coordinate systems in the small tubular neighborhoods of the heteroclinic orbits. Then, we get the Poincaré maps and bifurcation equations by means of the improved method.

Consider the *C* ^*r*^ system

(1) $$\begin{array}{c} \dot{z}=f\left(z\right), \end{array}$$

where *r* ≥ 4, *z* ∈ *R* ^*m*+*n*^. We assume the following.

(H1) Hyperbolic assumption: *z* = *p*_*i*_ are hyperbolic critical points of (1), *i* = 1,2. The stable manifold *W*_*p*_*i*__^*s*^ and the unstable manifold *W*_*p*_*i*__^*u*^ of *p*_*i*_ are *m*-dimensional and *n*-dimensional, respectively. Moreover, −*ρ*_*i*_^1^ and *λ*_*i*_^1^ are the simple real eigenvalues of *D*_*z*_ *f*(*p*_*i*_) such that any other eigenvalue *σ* of *D*_*z*_ *f*(*p*_*i*_) satisfies either *Reσ* < −*ρ*_*i*_^0^ < −*ρ*_*i*_^1^ < 0 or 0 < *λ*_*i*_^1^ < *λ*_*i*_^0^ < *Reσ*, where *ρ*_*i*_^0^ and *λ*_*i*_^0^ are some positive constants.

(H2) Nondegeneration: system (1) has a heteroclinic loop Γ = Γ_1_ ∪ Γ_2_, where Γ_*i*_ = {*z* = *r*_*i*_(*t*) : *t* ∈ *R*}, *r*_*i*_(+*∞*) = *r*_*i*+1_(−*∞*) = *p*_*i*+1_, *r*_3_(*t*) = *r*_1_(*t*), *p*_3_ = *p*_1_. dim⁡(*T*_*r*_*i*_(*t*)_ *W*_*p*_*i*__^*u*^∩*T*_*r*_*i*_(*t*)_ *W*_*p*_*i*+1__^*s*^) = 1.

(H3) Strong inclination: lim⁡_*x*→+*∞*_(*T*_*r*_*i*_(*t*)_ *W*_*p*_*i*__^*u*^ + *T*_*r*_*i*_(*t*)_ *W*_*p*_*i*+1__^*s*^) = *T*_*p*_*i*+1__ *W*_*p*_*i*+1__^*uu*^ ⊕ *T*_*p*_*i*+1__ *W*_*p*_*i*+1__^*s*^, lim⁡_*x*→−*∞*_(*T*_*r*_*i*_(*t*)_ *W*_*p*_*i*__^*u*^ + *T*_*r*_*i*_(*t*)_ *W*_*p*_*i*+1__^*s*^) = *T*_*p*_*i*__ *W*_*p*_*i*__^*u*^ ⊕ *T*_*p*_*i*__ *W*_*p*_*i*__^*ss*^, where *W*_*p*_*i*__^*uu*^ and *W*_*p*_*i*__^*ss*^ are the strong unstable manifolds and the strong stable manifolds, respectively, *T*_*p*_*i*__ *W*_*p*_*i*__^*uu*^ is the generalized eigenspace corresponding to all the eigenvalues with larger real part than *λ*_*i*_^0^, and *T*_*p*_*i*__ *W*_*p*_*i*__^*ss*^ is the generalized eigenspace corresponding to all the eigenvalues with smaller real part than −*ρ*_*i*_^0^. Denote $e_{i}^{\pm }=\lim_{t\to \mp \infty }{\dot{r}}_{i}(t)/\left|{\dot{r}}_{i}(t)\right|$, where *e*_*i*_^+^ ∈ *T*_*p*_*i*__ *W*_*p*_*i*__^*u*^ and *e*_*i*_^−^ ∈ *T*_*p*_*i*+1__ *W*_*p*_*i*+1__^*s*^ are unit eigenvectors corresponding to *λ*_*i*_^1^ and −*ρ*_*i*+1_^1^, respectively. Furthermore, span⁡(*T*_*p*_*i*__ *W*_*p*_*i*__^*uu*^, *e*_*i*_^+^) = *T*_*p*_*i*__ *W*_*p*_*i*__^*u*^, span⁡(*T*_*p*_*i*__ *W*_*p*_*i*__^*ss*^, *e*_*i*+1_^−^) = *T*_*p*_*i*__ *W*_*p*_*i*__^*s*^.

Now, we consider the following *C* ^*r*^ system:

(2) $$\begin{array}{c} \dot{z}=f\left(z\right)+g\left(z,\mu \right), \end{array}$$

where *μ* ∈ *R* ^*l*^, *l* ≥ 2, 0 ≤ |*μ*| ≪ 1, *g*(*p*_*i*_, *μ*) = *g*(*z*, 0) = 0, *i* = 1,2.

## 2. Local Coordinate Systems

Suppose that *U*_*i*_ is a sufficiently small neighborhood of *z* = *p*_*i*_ and (H1)~(H3) hold; then, for |*μ*| small enough, there exists a *C* ^*r*^ transformation such that system (2) has the following form in *U*_*i*_, respectively:

(3) x˙=[λi1(μ)+⋯]x+h.o.t.,y˙=[−ρi1(μ)+⋯]y+h.o.t.,u˙=[Bi1(μ)+⋯]u+h.o.t.,v˙=[−Bi2(μ)+⋯]v+h.o.t.,

where *z* = (*x*,*y*,u*,v*)*, *x* ∈ *R* ^1^, *y* ∈ *R* ^1^, *u* ∈ *R* ^*n*−1^, *v* ∈ *R* ^*m*−1^, ∗ means transposition, *λ*_*i*_^1^(0) = *λ*_*i*_^1^, *ρ*_*i*_^1^(0) = *ρ*_*i*_^1^, *Reσ*(*B*_*i*_^1^(0)) > *λ*_*i*_^0^, and *Reσ*(−*B*_*i*_^2^(0)) < −*ρ*_*i*_^0^. Moreover, we suppose

(4) Wpiu={z:y=0,v=0},Wpis={z:x=0,u=0},Wpiuu={z:x=x(u),y=0,v=0},Wpiss={z:x=0,u=0,y=y(v)},Γ∩Wpiu={z:u=u(x),y=0,v=0},Γ∩Wpis={z:x=0,u=0,v=v(y)},

where $x\left(0\right)=\dot{x}\left(0\right)=0$, $y\left(0\right)=\dot{y}\left(0\right)=0$, $u\left(0\right)=\dot{u}\left(0\right)=0$, $v\left(0\right)=\dot{v}\left(0\right)=0$.

Let *r*_*i*_(*t*) = (*r*_*i*_^*x*^(*t*),*r*_*i*_^*y*^(*t*),(*r*_*i*_^*u*^(*t*))*,(*r*_*i*_^*v*^(*t*))*)*; we can select some times *T*_*i*_^0^ and *T*_*i*_^1^ such that *r*_*i*_(−*T*_*i*_^0^) = (*δ*,0,*δ*_u_*,0*)*, *r*_*i*_(*T*_*i*_^1^) = (0,*δ*,0*,*δ*_v_*)*, where *δ* is small enough such that {(*x*,*y*,u*,v*)* : |*x*|, |*y*|, |*u*|, |*v*| < 2*δ*} ⊂ *U*_*i*_, |*δ*_*u*_| = *O*(*δ* ^*ω*^), |*δ*_*v*_| = *O*(*δ* ^*ω*^), *ω* = min⁡{*Reσ*(*B*_*i*_^2^(*μ*))/*ρ*_*i*_^1^(*μ*), *Reσ*(*B*_*i*_^1^(*μ*))/*λ*_*i*_^1^(*μ*)} > 1.

Consider the linear variational system and its adjoint system

(5) z˙=Df(ri(t))z,

(6) ϕ˙=−(Df(ri(t)))∗ϕ.

By [8, 9, 11–13], system (5) and (6) have exponential dichotomies in *R* ^+^ and *R* ^−^. It follows from [4] that system (5) has a fundamental solution matrix *Z*_*i*_(*t*) = (*z*_*i*_^1^(*t*), *z*_*i*_^2^(*t*), *z*_*i*_^3^(*t*), *z*_*i*_^4^(*t*)), satisfying

(7) zi1(t)∈(Tri(t)Wpiu∪Tri(t)Wpi+1s)c,zi2(t)=−r˙i(t)|r˙iy(Ti1)|∈Tri(t)Wpiu∩Tri(t)Wpi+1s,zi3(t)(zi3,1(t),…,zi3,n−1(t))∈Tri(t)Wpiu∩(Tri(t)Wpi+1s)c=Tri(t)Wpiuu,zi4(t)(zi4,1(t),…,zi4,m−1(t))∈(Tri(t)Wpiu)c∩Tri(t)Wpi+1s=Tri(t)Wpi+1ss,Zi(−Ti0)=(wi11wi210wi41wi1200wi42wi13wi23Iwi43000wi44),Zi(Ti1)=(10wi31001wi32000wi330wi14wi24wi34I),

where *w*_*i*_^21^ < 0, *w*_*i*_^12^ ≠ 0, ||*w*_*i*_^33^|| ≠ 0, ||*w*_*i*_^44^|| ≠ 0, and |*w*_*i*_^1*j*^(*w*_*i*_^12^)^−1^| ≪ 1, *j* ≠ 2; |*w*_*i*_^2*j*^(*w*_*i*_^21^)^−1^| ≪ 1, *j* = 3,4; |*w*_*i*_^3*j*^(*w*_*i*_^33^)^−1^| ≪ 1, *j* ≠ 3; |*w*_*i*_^4*j*^(*w*_*i*_^44^)^−1^| ≪ 1, *j* ≠ 4 for *δ* small enough.

Denote Φ_*i*_(*t*) = (*ϕ*_*i*_^1^(*t*), *ϕ*_*i*_^2^(*t*), *ϕ*_*i*_^3^(*t*), *ϕ*_*i*_^4^(*t*)) = (*Z*_*i*_^−1^(*t*))*; then Φ_*i*_(*t*) is a fundamental solution matrix of (6). It is well known that *ϕ*_*i*_^1^(*t*) is bounded and tends to zero exponentially as *t* → ±*∞* [8, 9, 12].

Denote Δ_*i*_ = *w*_*i*_^12^/|*w*_*i*_^12^|. Γ_*i*_ is nontwisted if Δ_*i*_ = 1 and twisted if Δ_*i*_ = −1. In this paper, we study the bifurcation problems under the nontwisted condition.

Let *z*_*i*_^1^(*t*), *z*_*i*_^2^(*t*), *z*_*i*_^3^(*t*), *z*_*i*_^4^(*t*) be the local coordinate systems along Γ_*i*_ in the tubular neighborhood of Γ_*i*_.

## 3. Poincaré Maps and Bifurcation Equations

Denote *h*_*i*_(*t*) = *r*_*i*_(*t*) + *Z*_*i*_(*t*)*N*_*i*_, *N*_*i*_ = (*n*_*i*_^1^,0,(*n*_*i*_^3^)*,(*n*_*i*_^4^)*)*, *n*_*i*_^3^ = (*n*_*i*_^3,1^,…,*n*_*i*_^3,*n*−1^)*, *n*_*i*_^4^ = (*n*_*i*_^4,1^,…,*n*_*i*_^4,*m*−1^)*. Define the Poincaré sections of Γ_*i*_ at *t* = −*T*_*i*_^0^ and *t* = *T*_*i*_^1^ by

**Figure 1:**
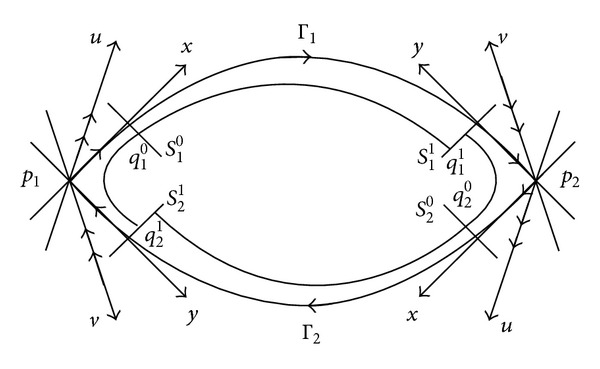


(8) Si0={z=hi(−Ti0):|x|,|y|,|u|,|v|<2δ},Si1={z=hi(Ti1):|x|,|y|,|u|,|v|<2δ},

respectively, where *δ* is small enough such that *S*_*i*_^0^ ⊂ *U*_*i*_, *S*_*i*_^1^ ⊂ *U*_*i*+1_, *U*_3_ = *U*_1_ (Figure 1).

Now, we set up the Poincaré map *F*_*i*_ = *F*_*i*_^1^∘*F*_*i*_^0^: *S*_*i*−1_^1^ ↦ *S*_*i*_^1^, where *F*_*i*_^0^: *q*_*i*−1_^1^ ∈ *S*_*i*−1_^1^ ↦ *q*_*i*_^0^ ∈ *S*_*i*_^0^, *F*_*i*_^1^: *q*_*i*_^0^ ∈ *S*_*i*_^0^ ↦ *q*_*i*_^1^ ∈ *S*_*i*_^1^, *S*_0_^1^ = *S*_2_^1^, and *q*_0_^1^ = *q*_2_^1^. Here, *F*_*i*_^1^ is constructed from the flow of (2) in the small tubular neighborhood of Γ_*i*_; *F*_*i*_^0^ is induced by the flow of the linear approximate system of (3) in the small neighborhood *U*_*i*_ of *p*_*i*_.

Denote

(9) qi0=(xi0,yi0,(ui0)∗,(vi0)∗)∗=ri(−Ti0)+Zi(−Ti0)Ni0,qi1=(xi1,yi1,(ui1)∗,(vi1)∗)∗=ri(Ti1)+Zi(Ti1)Ni1,

where *N*_*i*_^0^ = (*n*_*i*_^0,1^,0,(*n*_*i*_^0,3^)*,(*n*_*i*_^0,4^)*)*, *N*_*i*_^1^ = (*n*_*i*_^1,1^,0,(*n*_*i*_^1,3^)*,(*n*_*i*_^1,4^)*)*.

By the expressions of *Z*_*i*_(−*T*_*i*_^0^) and *Z*_*i*_(*T*_*i*_^1^), we get that the relations of the two kinds coordinates of *q*_*i*_^*j*^(*j* = 0,1) are *x*_*i*_^0^ ≈ *δ*, *y*_*i*_^1^ ≈ *δ*, and

(10) ni0,1=(wi12)−1[yi0−wi42(wi44)−1vi0],ni0,3=ui0−δu+[wi11wi23(wi21)−1−wi13](wi12)−1yi0 +{wi13(wi12)−1wi42−wi43−wi23(wi21)−1    ×[wi11(wi12)−1wi42−wi41]}(wi44)−1vi0,ni0,4=(wi44)−1vi0,ni1,1=xi1−wi31(wi33)−1ui1,ni1,3=(wi33)−1ui1,ni1,4=−wi14xi1+(wi14wi31+wi24wi32−wi34)(wi33)−1ui1 +vi1−δv.

First, we built the map *F*_*i*_^1^ : *S*_*i*_^0^ ↦ *S*_*i*_^1^, *q*_*i*_^0^ ↦ *q*_*i*_^1^. We make a coordinate transformation *z* = *h*_*i*_(*t*), *t* ∈ [−*T*_*i*_^0^, *T*_*i*_^1^] and substitute it into (2). By ${\dot{r}}_{i}\left(t\right)=f\left(r_{i}\left(t\right)\right)$, ${\dot{Z}}_{i}\left(t\right)=Df\left(r_{i}\left(t\right)\right)Z_{i}\left(t\right)$ and Φ_i_*(*t*)*Z*_*i*_(*t*) = *I*; (2) becomes

(11) $$\begin{array}{c} {\dot{n}}_{i}^{j}={\phi_{i}^{j}}^{*}\left(t\right)g_{\mu }\left(r_{i}\left(t\right),0\right)\mu +\text{h}.\text{o}.\text{t}.,\;j=1,3,4. \end{array}$$

Thus, we define *F*_*i*_^1^ : *S*_*i*_^0^ ↦ *S*_*i*_^1^, *q*_*i*_^0^ ↦ *q*_*i*_^1^ as

(12) $$\begin{array}{c} n_{i}^{j}\left(T_{i}^{1}\right)=n_{i}^{j}\left(-T_{1}^{0}\right)+M_{i}^{j}\mu +\text{h}.\text{o}.\text{t}.,\;j=1,3,4. \end{array}$$

That is,

(13) $$\begin{array}{c} n_{i}^{1,j}=n_{i}^{0,j}+M_{i}^{j}\mu +\text{h}.\text{o}.\text{t}.,\;j=1,3,4, \end{array}$$

where *M*_*i*_^*j*^ = ∫_−*T*_*i*_^0^_^*T*_*i*_^1^^ *ϕ*_*i*_^j^*(*t*)*g*_*μ*_(*r*_*i*_(*t*), 0)*dt*, *i* = 1,2, *j* = 1,3, 4, are called the Melnikov vectors.

By [7–9, 14], *M*_*i*_^*j*^ = ∫_−*T*_*i*_^0^_^*T*_*i*_^1^^ *ϕ*_*i*_^j^*(*t*)*g*_*μ*_(*r*_*i*_(*t*), 0)*dt* = ∫_−*∞*_^+*∞*^ *ϕ*_*i*_^j^*(*t*)*g*_*μ*_(*r*_*i*_(*t*), 0)*dt*, *i* = 1,2, *j* = 1,3, 4.

Second, we built the map *F*_*i*_^0^ : *S*_*i*−1_^1^ ↦ *S*_*i*_^0^, *q*_*i*−1_^1^ ↦ *q*_*i*_^0^, *S*_0_^1^ = *S*_2_^1^, *q*_0_^1^ = *q*_2_^1^. Without loss of generality, we assume *ρ*_*i*_^1^ ≥ *λ*_*i*_^1^. Let *τ*_*i*_ be the flying time from *q*_*i*−1_^1^ to *q*_*i*_^0^; we say *s*_*i*_ = *e* ^−*λ*_*i*_^1^(*μ*)*τ*_*i*_^ is* Silnikov* time. By (3) and neglecting the higher order terms, we have

(14) xi−11≈sixi0,  yi0≈siρi1(μ)/λi1(μ)yi−11,ui−11≈siBi1(μ)/λi1(μ)ui0,  vi0≈siBi2(μ)/λi1(μ)vi−11.

Here, (*s*_*i*_, *u*_*i*_^0^, *v*_*i*−1_^1^) is called the Silnikov coordinate.

Last, it follows from (10)~(14) that we get the expression of Poincaré map *F*_*i*_ = *F*_*i*_^1^∘*F*_*i*_^0^ as follows:

(15) ni1,1=(wi12)−1δsiρi1(μ)/λi1(μ)+Mi1μ+h.o.t.,ni1,3=ui0−δu+[wi11wi23(wi21)−1−wi13](wi12)−1δsiρi1(μ)/λi1(μ)   +Mi3μ+h.o.t.,ni1,4=(wi44)−1siBi2(μ)/λi1(μ)vi−11+Mi4μ+h.o.t.

Denote *G*_*i*_(*q*_*i*−1_^1^) = (*G*_*i*_^1^, *G*_*i*_^3^, *G*_*i*_^4^) = *F*_*i*_(*q*_*i*−1_^1^) − *q*_*i*_^1^. By (10) and (15), we get the successor function *G*_*i*_ as follows:

(16) Gi1=δ[(wi12)−1siρi1(μ)/λi1(μ)−si+1]+Mi1μ+h.o.t.,Gi3=ui0−δu+[wi11wi23(wi21)−1−wi13](wi12)−1δsiρi1(μ)/λi1(μ)   −(wi33)−1si+1Bi+11(μ)/λi+11(μ)ui+10+Mi3μ+h.o.t.,Gi4=−vi1+δv+wi14δsi+1+(wi44)−1siBi2(μ)/λi1(μ)vi−11   +Mi4μ+h.o.t.

Let

(17) $$\begin{array}{c} \left(G_{1}^{1},G_{1}^{3},G_{1}^{4},G_{2}^{1},G_{2}^{3},G_{2}^{4}\right)=0. \end{array}$$

Equation (17) is called the bifurcation equation.

## 4. The Preservations of Heteroclinic Orbits and Bifurcations of Homoclinic Loops

In this section, we discuss the nontwisted heteroclinic loop with resonant eigenvalues. Without loss of generality, we assume the following.

(H4) Resonant condition: *β*_1_ = *ρ*_1_^1^/*λ*_1_^1^ > 1, *β*_2_ = *ρ*_2_^1^/*λ*_2_^1^ = 1.

Let *β*_*i*_(*μ*) = *ρ*_*i*_^1^(*μ*)/*λ*_*i*_^1^(*μ*), *β*_*i*_ = *β*_*i*_(0), *ρ*_2_^1^(*μ*) = (1 + *α*(*μ*))*λ*_2_^1^(*μ*). By (H4) and the continuity of function, we get *β*_1_(*μ*) > 1, *β*_2_(*μ*) = 1 + *α*(*μ*), |*α*(*μ*)| ≪ 1, |*α*(0)| = 0, and *β*_1_(*μ*)*β*_2_(*μ*) > 1, for |*μ*| = 1.

Consider the solution of (17). Clearly, by the implicit function theorem, the equation (*G*_1_^3^, *G*_1_^4^, *G*_2_^3^, *G*_2_^4^) = 0 has a unique solution *u*_*i*_^0^ = *u*_*i*_^0^(*s*_1_, *s*_2_, *μ*), *v*_*i*_^1^ = *v*_*i*_^1^(*s*_1_, *s*_2_, *μ*), *i* = 1,2. Substituting it into (*G*_1_^1^, *G*_2_^1^) = 0, we have

(18) δ[(w112)−1s1β1(μ)−s2]+M11μ+h.o.t.=0,δ[(w212)−1s21+α(μ)−s1]+M21μ+h.o.t.=0.

Equation (18) is equivalent to

(19) s1=(w212)−1s21+α(μ)+δ−1M21μ+h.o.t.,s2=(w112)−1s1β1(μ)+δ−1M11μ+h.o.t.

Now, we study the existence of heteroclinic orbits and the 1-homoclinic bifurcations. Denote

(20) R12={μ:M11μ>0,w212M21μ<0,|μ|≪1},R21={μ:M21μ>0,w112M11μ<0,|μ|≪1}.

**Theorem 1**

Suppose that hypotheses (H1)~(H4) are valid, and rank (*M*_1_^1^, *M*_2_^1^) = 2; then one has the following.

(i) There exists a (*l* − 1)-dimensional surface *L*_*i*_ with a normal vector *M*_*i*_^1^ at *μ* = 0 such that (2) has a heteroclinic orbit joining *p*_1_ and *p*_2_ near Γ_*i*_ if and only if *μ* ∈ *L*_*i*_, |*μ*| ≪ 1, where *i* = 1,2. Moreover, (2) has a 1-heteroclinic loop near Γ if and only if *μ* ∈ *L*_12_ = *L*_1_∩*L*_2_ and |*μ*| ≪ 1, where *L*_12_ is (*l* − 2)-dimensional surface and 0 ∈ *L*_12_. That is, heteroclinic loop Γ is persistent.

(ii) There exists a (*l* − 1)-dimensional surface *L*_1_^2^ ⊂ *R*_1_^2^ which is tangent to *L*_2_(*L*_1_) at *μ* = 0 as *α*(*μ*) > 0  (*α*(*μ*) < 0), such that (2) has a unique 1-homoclinic loop Γ_1_^2^ homoclinic to *p*_1_ near Γ as *μ* ∈ *R*_1_^2^∩*L*_1_^2^ and |*μ*| ≪ 1. Meanwhile, there also exists a (*l* − 1)-dimensional surface *L*_2_^1^ ⊂ *R*_2_^1^ which is tangent to *L*_1_ at *μ* = 0 such that (2) has a unique 1-homoclinic loop Γ_2_^1^ homoclinic to *p*_2_ near Γ as *μ* ∈ *R*_2_^1^∩*L*_2_^1^ and |*μ*| ≪ 1.

**Proof**

(i) If (19) has a solution *s*_1_ = *s*_2_ = 0, then, we have

(21) $$\begin{array}{c} M_{i}^{1}\mu +\text{h}.\text{o}.\text{t}.=0,\;i=1,2. \end{array}$$

If *M*_*i*_^1^ ≠ 0, then, there exists a (*l* − 1)-dimensional surface *L*_*i*_ defined by (21) with a normal vector *M*_*i*_^1^ at *μ* = 0 such that the *i*th equation of (19) has a solution *s*_1_ = *s*_2_ = 0 as *μ* ∈ *L*_*i*_ and |*μ*| ≪ 1. That is, Γ_*i*_ is persistent.

If rank (*M*_1_^1^, *M*_2_^1^) = 2, then there exists a (*l* − 2)-dimensional surface *L*_12_ = *L*_1_∩*L*_2_ such that (19) has a solution *s*_1_ = *s*_2_ = 0 as *μ* ∈ *L*_12_ and |*μ*| ≪ 1. That is, Γ is persistent.

(ii) If (19) has a solution *s*_1_ = 0, *s*_2_ > 0, then, we have

(22) $$\begin{array}{c} s_{2}=\delta^{-1}M_{1}^{1}\mu +\text{h}.\text{o}.\text{t}.,\\ {\left(\delta^{-1}M_{1}^{1}\mu +\text{h}.\text{o}.\text{t}.\right)}^{1+\alpha (\mu )}=-\delta^{-1}w_{2}^{12}M_{2}^{1}\mu +\text{h}.\text{o}.\text{t}. \end{array}$$

If *M*_1_^1^ *μ* > 0, *w*_2_^12^ *M*_2_^1^ *μ* < 0, then, (22) defined a (*l* − 1)-dimensional surface *L*_1_^2^ with a normal vector *M*_2_^1^(*M*_1_^1^) at *μ* = 0 as *α*(*μ*) > 0  (*α*(*μ*) < 0), such that (19) has a solution *s*_1_ = 0, *s*_2_ > 0 as *μ* ∈ *L*_1_^2^ and |*μ*| ≪ 1. That is, the system (2) has a 1-homoclinic orbit to *p*_1_ near Γ for *μ* ∈ *L*_1_^2^ and |*μ*| ≪ 1.

(iii) If (19) has a solution *s*_1_ > 0, *s*_2_ = 0, then, we have

(23) $$\begin{array}{c} s_{1}=\delta^{-1}M_{2}^{1}\mu +\text{h}.\text{o}.\text{t}.,\\ {\left(\delta^{-1}M_{2}^{1}\mu +\text{h}.\text{o}.\text{t}.\right)}^{\beta_{1}(\mu )}=-\delta^{-1}w_{1}^{12}M_{1}^{1}\mu +\text{h}.\text{o}.\text{t}. \end{array}$$

If *M*_2_^1^ *μ* > 0, *w*_1_^12^ *M*_1_^1^ *μ* < 0, then, (23) defined a (*l* − 1)-dimensional surface *L*_2_^1^ with a normal vector *M*_1_^1^ at *μ* = 0 such that (19) has a solution *s*_1_ > 0, *s*_2_ = 0 as *μ* ∈ *L*_2_^1^ and |*μ*| ≪ 1. That is, the system (2) has a 1-homoclinic orbit to *p*_2_ near Γ for *μ* ∈ *L*_2_^1^ and |*μ*| ≪ 1.

## 5. The Periodic Orbits Bifurcations

Next, we consider the bifurcation problems of 1-periodic orbits near Γ. In other words, we study the solutions of (19) satisfying *s*_1_ > 0, *s*_2_ > 0. We assume the following.

(H5) Nontwisted condition: Δ_1_ = Δ_2_ = 1.

Obviously, if (H5) holds, we have

(24) $$\begin{array}{c} R_{1}^{2}=\left\{\mu :M_{1}^{1}\mu >0,M_{2}^{1}\mu <0,\left|\mu \right|\ll 1\right\},\\ R_{2}^{1}=\left\{\mu :M_{1}^{1}\mu <0,M_{2}^{1}\mu >0,\left|\mu \right|\ll 1\right\}. \end{array}$$

At first, we consider the bifurcations in *R*_1_^2^. From (19), we get

(25) (s1β1(μ)+δ−1w112M11μ+h.o.t.)1+α(μ) =(w112)1+α(μ)w212(s1−δ−1M21μ+h.o.t.).

Denote *V*_1_(*s*_1_) and *N*_1_(*s*_1_) are the left and right hands of (25), respectively; then, we have the following.

**Lemma 2**

Suppose that hypotheses (H1)~(H5) are valid; then *V*_1_(*s*_1_) is tangent to *N*_1_(*s*_1_) at some point *s*_1_ satisfying 0 < *s*_1_, |*μ*| ≪ 1 if and only if *μ* ∈ *R*_1_^2^, *α*(*μ*) > 0, |*M*_1_^1^ *μ*| ≪ |*M*_2_^1^ *μ*|, and

(26) δ−1M11μ =(−M11μw212M21μ)−1/α(μ)−(w112)−1  ×(−w112M11μ(1+α(μ))β1(μ)M21μ)β1(μ)/(β1(μ)−1)  +h.o.t.

**Proof**

*V*_1_(*s*_1_) is tangent to *N*_1_(*s*_1_) at some point *s*_1_ if and only if *V*_1_(*s*_1_) = *N*_1_(*s*_1_) and ${\dot{V}}_{1}\left(s_{1}\right)={\dot{N}}_{1}\left(s_{1}\right)$; that is,

(27) (s1β1(μ)+δ−1w112M11μ+h.o.t.)1+α(μ) =(w112)1+α(μ)w212(s1−δ−1M21μ+h.o.t.),(1+α(μ))β1(μ)s1β1(μ)−1(s1β1(μ)+δ−1w112M11μ+h.o.t.)α(μ) =(w112)1+α(μ)w212.

So

(28) $$\begin{array}{c} s_{1}={\left(-\frac{w_{1}^{12}M_{1}^{1}\mu }{\left(1+\alpha \left(\mu \right)\right)\beta_{1}(\mu )M_{2}^{1}\mu }\right)}^{1/\left(\beta_{1}(\mu )-1\right)}+\text{h}.\text{o}.\text{t}. \end{array}$$

Substituting (28) into the second expression of (27), we have that (26) holds.

Equation (28) means |*M*_1_^1^ *μ*||≪||*M*_2_^1^ *μ*||, and (26) means *μ* ∈ *R*_1_^2^ and *α*(*μ*) > 0.

If *M*_1_^1^ and *M*_2_^1^ are linearly independent, then there exists a (*l* − 1)-dimensional surface ${\tilde{L}}_{1}^{2}$ defined by (26) in the small neighborhood of *μ* = 0. It is easy to know that ${\tilde{L}}_{1}^{2}$∈*R*_1_^2^ is tangent to *L*_2_ for *α*(*μ*) > 0. By (21), (22), and (26), we get

(29) δ−1M11μ|L12=(−M11μw212M21μ)−(1/α(μ))+h.o.t.>(−M11μw212M21μ)−(1/α(μ))−(w112)−1 ×(−w112M11μ(1+α(μ))β1(μ)M21μ)β1(μ)/(β1(μ)−1) +h.o.t.=δ−1M11μ|L~12>0=δ−1M11μ|L1.

That is to say, ${\tilde{L}}_{1}^{2}$ is located in the open region between *L*_1_ and *L*_1_^2^. Thus, we have the following.

(1) For *α*(*μ*) > 0, we define the following three open sections in *R*_1_^2^: (*R*_1_^2^)_1_ is bounded by *L*_2_ and *L*_1_^2^, (*R*_1_^2^)_2_ is bounded by *L*_1_^2^ and ${\tilde{L}}_{1}^{2}$, and (*R*_1_^2^)_0_ is bounded by ${\tilde{L}}_{1}^{2}$ and *L*_1_, and they have nonempty intersection with *R*_1_^2^.

(2) For *α*(*μ*) < 0, we define the following two open sections in *R*_1_^2^: (*R*_1_^2^)_1_ is bounded by *L*_2_ and *L*_1_^2^ and (*R*_1_^2^)_0_ is bounded by *L*_1_^2^ and *L*_1_, and they have nonempty intersection with *R*_1_^2^.

Now, we consider the nonnegative solutions of *V*_1_(*s*_1_) = *N*_1_(*s*_1_) defined by (25). By *V*_1_(*s*_1_) = (*s*_1_^*β*_1_(*μ*)^+*δ* ^−1^ *w*_1_^12^ *M*_1_^1^ *μ*+h.o.t.)^1+*α*(*μ*)^, *N*_1_(*s*_1_) = (*w*_1_^12^)^1+*α*(*μ*)^ *w*_2_^12^(*s*_1_ − *δ* ^−1^ *M*_2_^1^ *μ* + h.o.t.), we get *V*_1_(0) = (*δ* ^−1^ *w*_1_^12^ *M*_1_^1^ *μ*+h.o.t.)^1+*α*(*μ*)^, *N*_1_(0) = (*w*_1_^12^)^1+*α*(*μ*)^ *w*_2_^12^(−*δ* ^−1^ *M*_2_^1^ *μ* + h.o.t.). From (22), *V*_1_(0) = *N*_1_(0) holds for *μ* ∈ *L*_1_^2^, |*μ*| ≪ 1.

By Lemma 2, it is easy to get the following conclusions.

**Figure 2:**
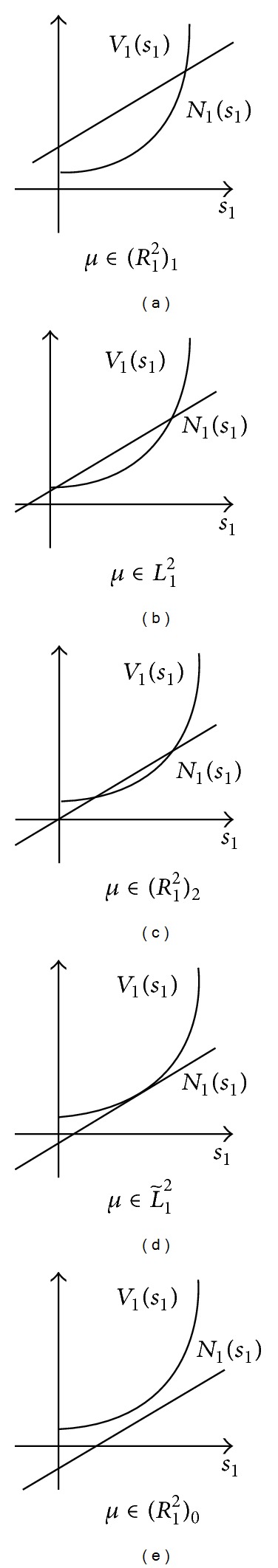


1. For *α*(*μ*) > 0, we have the following (see Figure 2).
2. If *μ* ∈ (*R*_1_^2^)_1_, then *V*_1_(*s*_1_) = *N*_1_(*s*_1_) has one small positive solution.
3. If *μ* ∈ *L*_1_^2^, then *V*_1_(*s*_1_) = *N*_1_(*s*_1_) has one small positive solution and one zero solution.
4. If *μ* ∈ (*R*_1_^2^)_2_, then *V*_1_(*s*_1_) = *N*_1_(*s*_1_) has two small different positive solutions.
5. If $\mu \in {\tilde{L}}_{1}^{2}$, then *V*_1_(*s*_1_) = *N*_1_(*s*_1_) has one small twofold positive solution.
6. If *μ* ∈ (*R*_1_^2^)_0_, then *V*_1_(*s*_1_) = *N*_1_(*s*_1_) does not have any small nonnegative solutions.
7. For *α*(*μ*) < 0, we have the following.
8. If *μ* ∈ (*R*_1_^2^)_1_, then *V*_1_(*s*_1_) = *N*_1_(*s*_1_) has one small positive solution.
9. If *μ* ∈ *L*_1_^2^, then *V*_1_(*s*_1_) = *N*_1_(*s*_1_) has one zero solution.
10. If *μ* ∈ (*R*_1_^2^)_0_, then *V*_1_(*s*_1_) = *N*_1_(*s*_1_) has not any small nonnegative solutions.

Thus, we have shown the following conclusions.

**Theorem 3**

Suppose that hypotheses (H1)~(H5) are valid, *μ* ∈ *R*_1_^2^, *α*(*μ*) > 0; then the following conclusions are true.

- System (2) has one simple 1-periodic orbit near Γ as *μ* ∈ (*R*_1_^2^)_1_.
- System (2) has one simple 1-periodic orbit and one 1-homoclinic loop homoclinic to *p*_1_ near Γ as *μ* ∈ *L*_1_^2^.
- System (2) has two simple 1-periodic orbits near Γ as *μ* ∈ (*R*_1_^2^)_2_.
- System (2) has a unique twofold 1-periodic orbit near Γ as $\mu \in {\tilde{L}}_{1}^{2}$.
- System (2) has not any 1-periodic orbits and 1-homoclinic loops near Γ as *μ* ∈ (*R*_1_^2^)_0_.

**Theorem 4**

Suppose that hypotheses (H1)~(H5) are valid, *μ* ∈ *R*_1_^2^, *α*(*μ*) < 0; then the following conclusions are true.

- System (2) has one simple 1-periodic orbit near Γ as *μ* ∈ (*R*_1_^2^)_1_.
- System (2) has one 1-homoclinic loop homoclinic to *p*_1_ near Γ as *μ* ∈ *L*_1_^2^.
- System (2) does not have any 1-periodic orbits and 1-homoclinic loops near Γ as *μ* ∈ (*R*_1_^2^)_0_.

Next, we consider the bifurcations in *R*_2_^1^. Similarly, from (19), we get

(30) (s21+α(μ)+δ−1w212M21μ+h.o.t.)β1(μ) =w112(w212)β1(μ)(s2−δ−1M11μ+h.o.t.).

Denote *V*_2_(*s*_2_) and *N*_2_(*s*_2_) are the left and right hands of (30), respectively; then one has the following.

**Lemma 5**

Suppose that hypotheses (H1)~(H5) are valid, then *V*_2_(*s*_2_) is tangent to *N*_2_(*s*_2_) at some point *s*_2_ satisfying 0 < *s*_2_, |*μ*| ≪ 1 if and only if *α*(*μ*) < 0, ||*M*_1_^1^ *μ*|| ≪ ||*M*_2_^1^ *μ*||, *μ* ∈ *R*_2_^1^ and

(31) δ−1M21μ =(−w112M11μM21μ)1/(β1(μ)−1)−(w212)−1  ×(−(1+α(μ))β1(μ)M11μw212M21μ)−(1+α(μ))/α(μ)  + h.o.t.

**Proof**

*V*_2_(*s*_2_) is tangent to *N*_2_(*s*_2_) at some point *s*_2_ if and only if *V*_2_(*s*_2_) = *N*_2_(*s*_2_) and ${\dot{V}}_{2}\left(s_{2}\right)={\dot{N}}_{2}\left(s_{2}\right)$; that is,

(32) (s21+α(μ)+δ−1w212M21μ+h.o.t.)β1(μ) =w112(w212)β1(μ)(s2−δ−1M11μ+h.o.t.),(1+α(μ))β1(μ)s2α(μ)(s21+α(μ)+δ−1w212M21μ+h.o.t.)β1(μ)−1 =w112(w212)β1(μ)+h.o.t.

The solution of (32) is

(33) $$\begin{array}{c} s_{2}={\left(-\frac{w_{2}^{12}M_{2}^{1}\mu }{\left(1+\alpha \left(\mu \right)\right)\beta_{1}\left(\mu \right)M_{1}^{1}\mu }\right)}^{1/\alpha (\mu )}+\text{h}.\text{o}.\text{t}. \end{array}$$

Substituting (33) into the second expression of (32), we obtain (31).

Equation (33) holds means |*M*_1_^1^ *μ*||≪||*M*_2_^1^ *μ*||, *α*(*μ*) < 0, or |*M*_2_^1^ *μ*||≪||*M*_1_^1^ *μ*||, *α*(*μ*) > 0. But, if |*M*_2_^1^ *μ*||≪||*M*_1_^1^ *μ*||, *α*(*μ*) > 0, then, by (31), we get that *δ* ^−1^ *M*_2_^1^ *μ* will be tented to *∞*. Last, *μ* ∈ *R*_2_^1^ is obvious.

If *M*_1_^1^ and *M*_2_^1^ are linearly independent, then there exists a (*l* − 1)-dimensional surface ${\tilde{L}}_{2}^{1}$ defined by (31) in the small neighborhood of *μ* = 0. It is easy to know that ${\tilde{L}}_{2}^{1}$ is tangent to *L*_1_ at *μ* = 0. By (21), (23), and (31), we get

(34) δ−1M21μ|L21=(−w112M11μM21μ)1/(β1(μ)−1)+h.o.t.>(−w112M11μM21μ)1/(β1(μ)−1)−(w212)−1 ×(−(1+α(μ))β1(μ)M11μw212M21μ)−(1+α(μ))/α(μ) +h.o.t.=δ−1M21μ|L~21>0=δ−1M21μ|L2.

That is, ${\tilde{L}}_{2}^{1}$ is located in the open region between *L*_2_ and *L*_2_^1^. Thus, we have the following.

(1) For *α*(*μ*) > 0, we define the following two open sections: (*R*_2_^1^)_1_ is bounded by *L*_1_ and *L*_2_^1^ and (*R*_2_^1^)_0_ is bounded by *L*_2_^1^ and *L*_2_, and they have nonempty intersection with *R*_2_^1^.

(2) For *α*(*μ*) < 0, we define the following three open sections: (*R*_2_^1^)_1_ is bounded by *L*_1_ and *L*_2_^1^, (*R*_2_^1^)_2_ is bounded by *L*_2_^1^ and ${\tilde{L}}_{2}^{1}$, and (*R*_2_^1^)_0_ is bounded by ${\tilde{L}}_{2}^{1}$ and *L*_2_, and they have nonempty intersection with *R*_2_^1^.

Now, we consider the nonnegative solutions of *V*_2_(*s*_2_) = *N*_2_(*s*_2_) defined by (30). By *V*_2_(*s*_2_) = (*s*_2_^1+*α*(*μ*)^+*δ* ^−1^ *w*_2_^12^ *M*_2_^1^ *μ*+h.o.t.)^*β*_1_(*μ*)^, *N*_2_(*s*_2_) = *w*_1_^12^(*w*_2_^12^)^*β*_1_(*μ*)^(*s*_2_ − *δ* ^−1^ *M*_1_^1^ *μ* + h.o.t.), we get *V*_2_(0) = (*δ* ^−1^ *w*_2_^12^ *M*_2_^1^ *μ*+h.o.t.)^*β*_1_(*μ*)^, *N*_2_(0) = *w*_1_^12^(*w*_2_^12^)^*β*_1_(*μ*)^(−*δ* ^−1^ *M*_1_^1^ *μ* + h.o.t.). From (23), *V*_2_(0) = *N*_2_(0) holds for *μ* ∈ *L*_2_^1^, |*μ*| ≪ 1.

By Lemma 5, it is easy to get the following conclusions.

1. For *α*(*μ*) > 0, we have the following.
   If *μ* ∈ (*R*_2_^1^)_1_, then *V*_2_(*s*_2_) = *N*_2_(*s*_2_) has one small positive solution.
   If *μ* ∈ *L*_2_^1^, then *V*_2_(*s*_2_) = *N*_2_(*s*_2_) has one zero solution.
   If *μ* ∈ (*R*_2_^1^)_0_, then *V*_2_(*s*_2_) = *N*_2_(*s*_2_) does not have any small nonnegative solutions.
2. For *α*(*μ*) < 0, we have the following.
   If *μ* ∈ (*R*_2_^1^)_1_, then *V*_2_(*s*_2_) = *N*_2_(*s*_2_) has one small positive solution.
   If *μ* ∈ *L*_2_^1^, then *V*_2_(*s*_2_) = *N*_2_(*s*_2_) has one small positive solution and one zero solution.
   If *μ* ∈ (*R*_2_^1^)_2_, then *V*_2_(*s*_2_) = *N*_2_(*s*_2_) has two different positive solutions.
   If $\mu \in {\tilde{L}}_{2}^{1}$, then *V*_2_(*s*_2_) = *N*_2_(*s*_2_) has one twofold positive solution.
   If *μ* ∈ (*R*_2_^1^)_0_, then *V*_2_(*s*_2_) = *N*_2_(*s*_2_) does not have any small nonnegative solutions.

Thus, we have shown the following conclusions.

**Theorem 6**

Suppose that hypotheses (H1)~(H5) are valid, *μ* ∈ *R*_2_^1^, *α*(*μ*) > 0; then the following conclusions are true.

- System (2) has one simple 1-periodic orbit near Γ as *μ* ∈ (*R*_2_^1^)_1_.
- System (2) has one 1-homoclinic loop homoclinic to *p*_2_ near Γ as *μ* ∈ *L*_2_^1^.
- System (2) does not have any 1-periodic orbits and 1-homoclinic loops near Γ as *μ* ∈ (*R*_2_^1^)_0_.

**Theorem 7**

Suppose that hypotheses (H1)~(H5) are valid, *μ* ∈ *R*_2_^1^, *α*(*μ*) < 0; then the following conclusions are true.

- System (2) has one simple 1-periodic orbit near Γ as *μ* ∈ (*R*_2_^1^)_1_.
- System (2) has one simple 1-periodic orbit and one 1-homoclinic loop homoclinic to *p*_2_ near Γ as *μ* ∈ *L*_2_^1^.
- System (2) has two simple 1-periodic orbits near Γ as *μ* ∈ (*R*_2_^1^)_2_.
- System (2) has a unique twofold 1-periodic orbit near Γ as $\mu \in {\tilde{L}}_{2}^{1}$.
- System (2) does not have any 1-periodic orbits and 1-homoclinic loops near Γ as *μ* ∈ (*R*_2_^1^)_0_.

Let *D*_1_^2^ be an open region that is bounded by *L*_1_ and *L*_2_; meanwhile, *D*_1_^2^∩{*μ* : *M*_1_^1^ *μ* > 0, *M*_2_^1^ *μ* > 0, |*μ*| ≪ 1} ≠ *∅*. Let *D*_2_^1^ be an open region that is bounded by *L*_2_ and *L*_1_; meanwhile, *D*_2_^1^∩{*μ* : *M*_1_^1^ *μ* < 0, *M*_2_^1^ *μ* < 0, |*μ*| ≪ 1} ≠ *∅*.

By (19), it is easy to see that (19) has an unique solution *s*_1_ > 0, *s*_2_ > 0 for *μ* ∈ *D*_1_^2^ and does not have any nonnegative solutions for *μ* ∈ *D*_2_^1^. So we get the following conclusions.

**Theorem 8**

Supposing that hypotheses (H1)~(H5) are valid, then

- system (2) has one simple 1-periodic orbit near Γ as *μ* ∈ *D*_1_^2^,
- system (2) has not any 1-periodic orbits, 1-homoclinic loops, and heteroclinic orbits near Γ as *μ* ∈ *D*_2_^1^.

**Figure 3:**
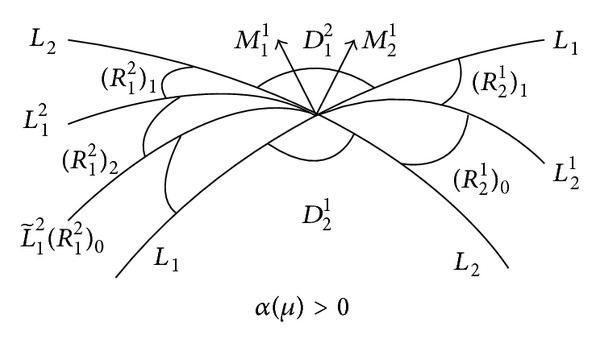


Combining Theorems 1~8, we get the bifurcation figures (see Figures 3 and 4).

## Figures and Tables

**Figure 4:**